# DAP3 Is Involved in Modulation of Cellular Radiation Response by RIG-I-Like Receptor Agonist in Human Lung Adenocarcinoma Cells

**DOI:** 10.3390/ijms22010420

**Published:** 2021-01-03

**Authors:** Yoshiaki Sato, Hironori Yoshino, Ikuo Kashiwakura, Eichi Tsuruga

**Affiliations:** Department of Radiation Science, Graduate School of Health Sciences, Hirosaki University, Hirosaki, Aomori 036-8564, Japan; h20gg702@hirosaki-u.ac.jp (Y.S.); ikashi@hirosaki-u.ac.jp (I.K.); tsuru@hirosaki-u.ac.jp (E.T.)

**Keywords:** retinoic acid-inducible gene-I-like receptor, ionizing radiation, radiosensitivity, mitochondria, death-associated protein 3, lung adenocarcinoma

## Abstract

Retinoic acid-inducible gene-I (RIG-I)-like receptors (RLRs) mediate anti-viral response through mitochondria. In addition, RLR activation induces anti-tumor effects on various cancers. We previously reported that the RLR agonist Poly(I:C)-HMW/LyoVec™ (Poly(I:C)) enhanced radiosensitivity and that cotreatment with Poly(I:C) and ionizing radiation (IR) more than additively increased cell death in lung adenocarcinoma cells, indicating that Poly(I:C) modulates the cellular radiation response. However, it remains unclear how mitochondria are involved in the modulation of this response. Here, we investigated the involvement of mitochondrial dynamics and mitochondrial ribosome protein death-associated protein 3 (DAP3) in the modulation of cellular radiation response by Poly(I:C) in A549 and H1299 human lung adenocarcinoma cell lines. Western blotting revealed that Poly(I:C) decreased the expression of mitochondrial dynamics-related proteins and DAP3. In addition, siRNA experiments showed that DAP3, and not mitochondrial dynamics, is involved in the resistance of lung adenocarcinoma cells to IR-induced cell death. Finally, we revealed that a more-than-additive effect of cotreatment with Poly(I:C) and IR on increasing cell death was diluted by DAP3-knockdown because of an increase in cell death induced by IR alone. Together, our findings suggest that RLR agonist Poly(I:C) modulates the cellular radiation response of lung adenocarcinoma cells by downregulating DAP3 expression.

## 1. Introduction

Mitochondria are essential organelles for regulating cellular functions, such as oxidative phosphorylation, cell death, and immune responses [1,2]. Mitochondria are highly dynamic organelles that undergo fusion and fission, referred to as mitochondrial dynamics [3]. These processes are regulated by several proteins [3], e.g., mitochondrial fusion is mainly regulated by mitofusin-1/2 (Mfn1/2) and optic atrophy protein 1 (OPA1), with the former involved in outer membrane fusion and the latter in inner membrane fusion, whereas dynamin-related protein 1 (Drp1) is the main protein that initiates mitochondrial fission. In addition, the mitochondria contain their own DNA and ribosomes that synthesize mitochondrial DNA (mtDNA)-encoded proteins [4]. These characteristics have been reported to be important in self-maintenance of mitochondrial functions in response to various stress conditions such as viral infection [5,6,7].

Retinoic acid-inducible gene-I (RIG-I)-like receptors (RLRs) are pattern-recognition receptors that recognize pathogen-associated molecular patterns in the cytosolic fraction. RLRs detect viral RNA and elicit anti-viral responses, such as the induction of type I interferons (IFNs) through the adaptor molecule mitochondrial anti-viral signaling protein located in the mitochondrial membrane [8,9]. Furthermore, recent studies have shown that RLR activation induces anti-tumor effects, including anti-tumor immunity and cell death in various cancer types, such as lung cancer [10,11,12]. Therefore, strategies for cancer therapy focusing on RLR activation have been studied [12,13,14].

Our previous report showed that the RLR agonist synthetic double-stranded RNA Poly(I:C)-HMW/LyoVec™ (Poly(I:C)) enhanced radiosensitivity and that cotreatment with Poly(I:C) and ionizing radiation (IR) exerted a more-than-additive effect for each treatment alone in inducing cell death in human lung adenocarcinoma cells [15]. These results indicate that Poly(I:C) modulates cellular radiation responses. However, it remains unknown how mitochondria are involved in the modulation of cellular radiation responses by Poly(I:C) in human lung adenocarcinoma cells.

Growing evidence has demonstrated that mitochondrial dynamics and mitochondrial ribosome proteins are involved in cellular responses to various stresses, including radiation and viral infection [5,16,17,18,19]. For example, it has been reported that mitochondrial fission-related proteins are involved in the radiosensitivity of EMT6 murine breast cancer cells [16]. In addition, mitochondrial dynamics are reported to regulate RLRs-mediated antiviral immune response [5]. Moreover, Kim et al. reported that Hepatitis C virus causes mitochondrial fission, which leads to evasion of apoptosis in huh-7 human hepatocellular carcinoma cells [17]. Among mitochondrial ribosome proteins, death-associated protein 3 (DAP3; mitochondrial ribosome protein S29) is known as a GTP-binding protein and a major positive mediator of cell death [18]. Conversely, Henning reported that overexpression of DAP3 conferred radioresistance to ataxia telangiectasia cells exhibiting high radiosensitivity [19]. Considering these findings, we hypothesized that the RLR agonist Poly(I:C) modulates the cellular radiation response by regulating mitochondrial dynamics or the mitochondrial ribosome protein DAP3. To address this hypothesis, we investigated the relationship between mitochondrial dynamics, DAP3, and the modulation of the cellular radiation response by Poly(I:C) in human lung adenocarcinoma cells.

The major findings of this study were as follows: (i) Poly(I:C) decreased the expression of mitochondrial dynamics-related proteins and DAP3 in human lung adenocarcinoma cells; (ii) DAP3 was involved in the resistance of lung adenocarcinoma cells to IR-induced cell death, whereas mitochondrial dynamics were not; (iii) a more-than-additive effect of cotreatment with Poly(I:C) and IR on increasing cell death was diluted by DAP3-knockdown because of an increase in cell death induced by IR alone. These findings suggest that the RLR agonist Poly(I:C) modulates cellular radiation response of lung adenocarcinoma cells by downregulating DAP3 protein expression.

## 2. Results

### 2.1. Expression of Mitochondrial Dynamics-Related Proteins and Mitochondrial Morphology in A549 Cells Treated with Poly(I:C) and/or IR

As shown in Figure S1A, the cell death in Poly(I:C)-treated A549 cells was increased with time. In addition, the effect of Poly(I:C) to increase IR-induced cell death occurred at around 48 h after cotreatment with Poly(I:C), and it was clearly observed at 72 h (Figure S1B). Therefore, to clarify the mechanisms by which Poly(I:C) modulates cellular radiation response, analyses were mainly performed at 48 or 72 h after the treatment with Poly(I:C) and/or IR in this study.

**Figure 1:**
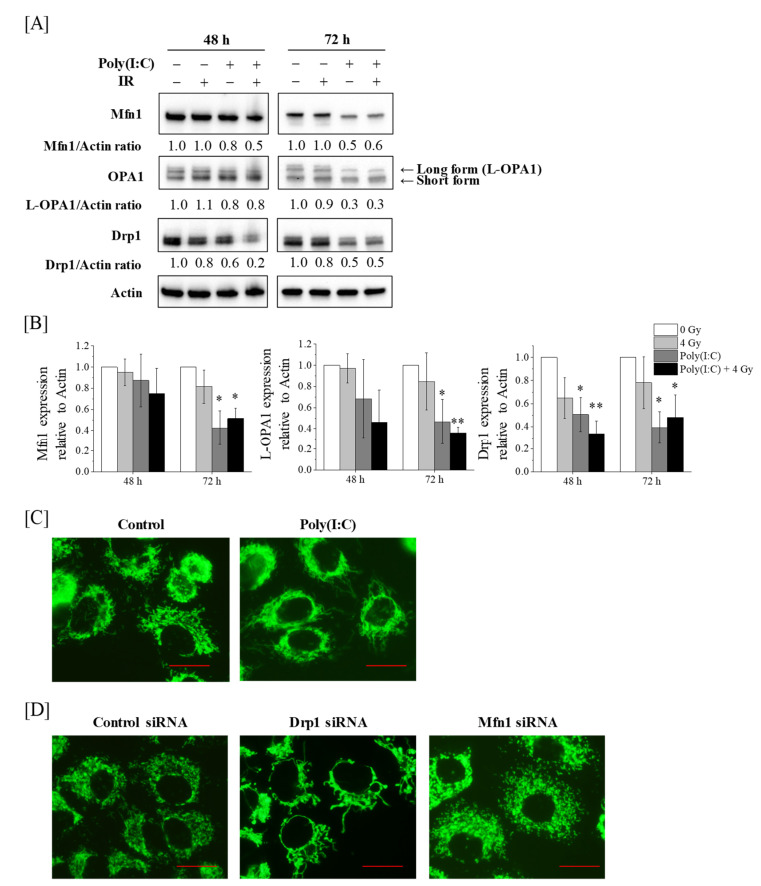
Effect of Poly(I:C)-HMW/LyoVec™ (Poly(I:C)) and/or ionizing radiation (IR) on mitochondrial dynamics in A549 cells. (**A**,**B**) A549 cells were incubated with Poly(I:C). After incubation for 1 h, the cells were irradiated with 4 Gy. After culturing for 48 or 72 h, the cells were harvested for western blotting. (**A**) Representative images of immunoblots are shown. Actin was used as a loading control. (**B**) The relative values of Mfn1/actin, L-OPA1/actin and Drp1/actin ratio are presented as mean ± SD of three independent experiments. For the Drp1 proteins, both bands were quantified together. One sample *t*-test was performed using the GraphPad QuickCalcs. * *p* < 0.05, ** *p* < 0.01 versus control. (**C**) A549 cells cultured for 72 h in the presence of Poly(I:C) were harvested for mitochondrial morphology analysis using the MitoTracker^TM^ Green FM. (**D**) A549 cells transfected with control, Drp1, or Mfn1 siRNA were cultured for 72 h and harvested for mitochondrial morphology analysis. Scale bar = 20 μm.

We initially analyzed the expression of mitochondrial dynamics-related proteins in A549 cells treated with Poly(I:C), IR, or both. As shown in Figure 1A,B, the expression of the mitochondria fission-related protein Drp1 was significantly lower in the cells treated with Poly(I:C) at 48 h and 72 h. Similarly, Poly(I:C) or cotreatment with Poly(I:C) and IR decreased the expression of mitochondrial fusion-related protein Mfn1 or fusion-competent long isoform OPA1 (L-OPA1), not short isoform OPA1 [20], at 72 h after the treatment, whereas this was not observed at 48 h after the treatment (Figure 1A,B).

**Figure 2:**
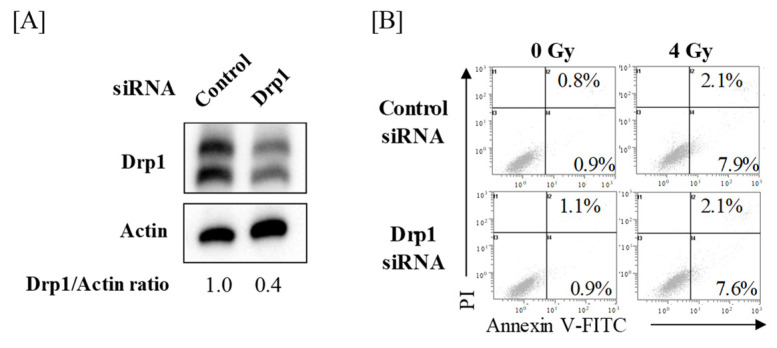
Effects of Drp1-knockdown on IR-induced cell death in A549 cells. (**A**) A549 cells transfected with control or Drp1 siRNA were harvested, and the Drp1 protein expression was analyzed by western blotting. Representative images of immunoblots are shown. Actin was used as a loading control. The relative values of Drp1/actin ratio are presented. For the Drp1 proteins, both bands were quantified together. (**B**) Drp1-knockdown A549 cells were treated with 4 Gy. After culturing for 72 h, the cells were harvested for cell death analysis using annexin V-FITC/propidium iodide (PI) staining. Representative cytograms of annexin V/PI staining are shown. The inset numbers indicate the fractions of annexin V+/PI− or annexin V+/PI+ cells.

As Poly(I:C) decreased the expression of mitochondrial dynamics-related proteins, we analyzed the mitochondrial morphology of A549 cells treated with Poly(I:C). As shown in Figure 1C, A549 cells treated with Poly(I:C) had elongated mitochondria when compared with the control cells. This morphology was similar to that of Drp1-knockdown A549 cells wherein Drp1 protein expression was decreased by transfection with siRNA-targeting Drp1 (Figure 1D and Figure 2A) but not to Mfn1-knockdown cells whose mitochondria were fragmented (Figure 1D and Figure S2A).

### 2.2. Effect of Drp1-Knockdown on IR-Induced Cell Death in A549 Cells

As Poly(I:C) decreased Drp1 expression prior to Mfn1 and L-OPA1 downregulation and as Poly(I:C)-treated A549 cells exhibited elongated mitochondria similar to that in Drp1-knockdown cells, we focused on Drp1. To investigate whether Drp1 is involved in IR-induced cell death, Drp1-knockdown A549 cells (Figure 2A) were irradiated with X-ray, followed by cell death analysis. Analysis of cell death using annexin V-FITC and propidium iodide (PI) staining revealed that there was no significant difference in relative cell death (sum of annexin V+/PI− and annexin V+/PI+ cells) between control and Drp1-knockdown cells after IR (Figure 2B).

### 2.3. Downregulation of DAP3 Protein Expression by Poly(I:C) in Human Lung Adenocarcinoma Cells

**Figure 3:**
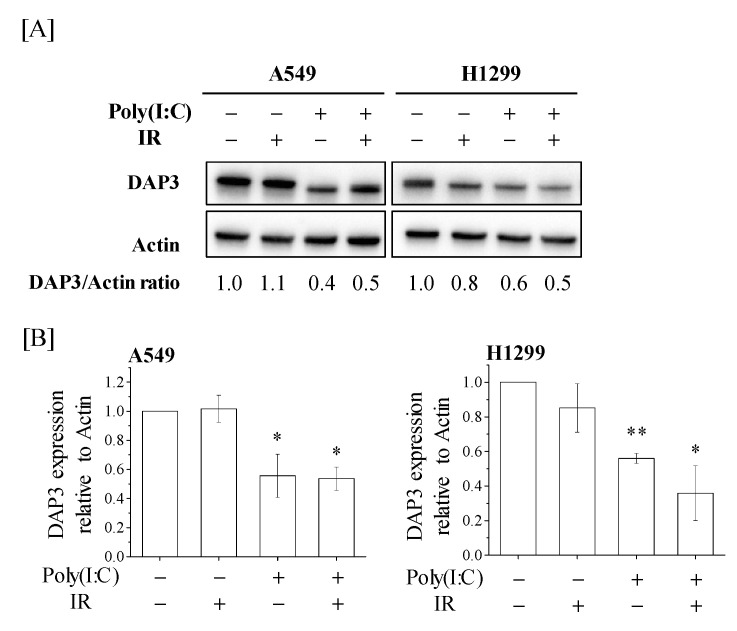
Death-associated protein 3 (DAP3) expression in human lung adenocarcinoma cells treated with Poly(I:C) and/or IR. (**A**,**B**) A549 and H1299 cells were incubated with Poly(I:C). After incubation for 1 h, the cells were irradiated with 4 Gy. After culturing for 72 h, the cells were harvested for western blotting. (**A**) Representative images of immunoblots are shown. Actin was used as a loading control. (**B**) The relative values of DAP3/actin ratio are presented as mean ± SD of three independent experiments. One sample *t*-test was performed using the GraphPad QuickCalcs. * *p* < 0.05, ** *p* < 0.01 versus control.

We then investigated DAP3 expression in A549 and H1299 human lung adenocarcinoma cells treated with Poly(I:C) and/or IR. As shown in Figure 3A, Poly(I:C) or cotreatment with Poly(I:C) and IR decreased DAP3 protein expression, and a significant decrease in DAP3 protein expression was observed in the Poly(I:C)-treated group as compared with the control group (Figure 3B).

### 2.4. Involvement of DAP3 in Radioresistance of Human Lung Adenocarcinoma Cells

**Figure 4:**
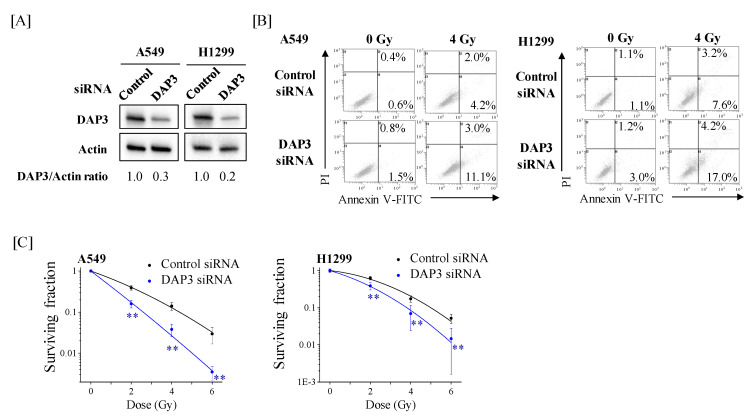
Effects of DAP3-knockdown on IR-induced cell death and radiosensitivity of human lung adenocarcinoma cells. (**A**) A549 and H1299 cells transfected with control or DAP3 siRNA were harvested, and DAP3 protein expression was analyzed by western blotting. Representative images of immunoblots are shown. Actin was used as a loading control. The relative values of DAP3/actin ratio are presented. (**B**) DAP3-knockdown A549 and H1299 cells were irradiated with 4 Gy. After culturing for 72 h, the cells were harvested for cell death analysis using annexin V-FITC/PI staining. Representative cytograms of annexin V/PI staining are shown. The inset numbers indicate the fractions of annexin V+/PI− or annexin V+/PI+ cells. (**C**) DAP3-knockdown A549 and H1299 cells were irradiated with X-rays. After a 20-h incubation, the cells were harvested and seeded in fresh media and further cultured until noticeable growth. The surviving fraction of A549 and H1299 cells is shown. Data are presented as mean ± SD of three independent experiments. ** *p* < 0.01 versus control siRNA.

We next examined the role of DAP3 in the radiation response of human lung adenocarcinoma cells using DAP3-knockdown cells (Figure 4A). Relative cell death in DAP3-knockdown cells following IR was higher than that in control cells (Figure 4B). Moreover, DAP3-knockdown markedly decreased the survival fraction in irradiated A549 and H1299 cells (Figure 4C). The radiation dose at which 10% of cells survived (D_10_) was reduced from 4.38 Gy in control cells to 2.59 Gy in DAP3-knockdown A549 cells. In H1299 cells, the D_10_ was reduced from 5.00 Gy in control cells to 3.81 Gy in DAP3-knockdown cells. Collectively, these results indicate that DAP3 is involved in radioresistance of human lung adenocarcinoma cells.

### 2.5. Involvement of DAP3 in the More-Than-Additive Effect of Cotreatment with Poly(I:C) and IR on Cell Death in Human Lung Adenocarcinoma Cells

**Figure 5:**
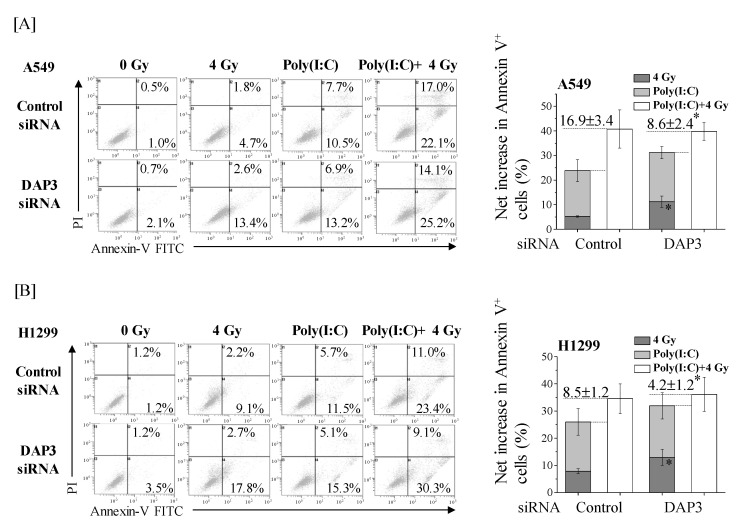
DAP3 is involved in the more-than-additive effect of cotreatment with Poly(I:C) and IR on induction of cell death. DAP3-knockdown A549 (**A**) and H1299 cells (**B**) were incubated with Poly(I:C). After incubation for 1 h, the cells were irradiated with 4 Gy. After culturing for 72 h, the cells were harvested for cell death assay using annexin V/PI staining. (**left**) Representative cytograms of annexin V/PI staining are shown. The inset numbers indicate the fractions of annexin V+/PI− or annexin V+/PI+ cells. (**right**) The results are presented as the net increase in the fraction of annexin V+ cells (the sum of annexin V+/PI− cells and annexin V+/PI+ cells). Data are presented as mean ± SD of three independent experiments. * *p* < 0.05 versus control siRNA.

We investigated whether DAP3 is involved in the more-than-additive increase in the death of human lung adenocarcinoma cells caused by cotreatment with Poly(I:C) and IR. In line with our recent report [15], the percentage of annexin V+ cells was higher in cotreated cells than in cells treated with IR or Poly(I:C) alone (Figure 5A(left)). The net increase in annexin V+ fraction was about 17% higher with cotreatment than the sum of fractions induced by IR and Poly(I:C) individually. Interestingly, the sum of annexin V+ fractions induced by IR and Poly(I:C) individually was increased by DAP3-knockdown, whereas no significant difference in the annexin V+ fraction of cotreated cells was observed between the control and DAP3-knockdown cells (Figure 5A(right)). As a result, the difference between that sum and the fraction of annexin V+ cells induced by cotreatment with Poly(I:C) and IR was significantly decreased to approximately 8% upon DAP3-knockdown (Figure 5A(right)). Similar to results in A549 cells, DAP3-knockdown significantly increased IR-induced cell death in H1299 cells, which diluted the more-than-additive effect of cotreatment on cell death from 8.5% to 4.2% (Figure 5B).

### 2.6. Post-Transcriptional Downregulation of DAP3 Expression by Poly(I:C) in A549 Cells

**Figure 6:**
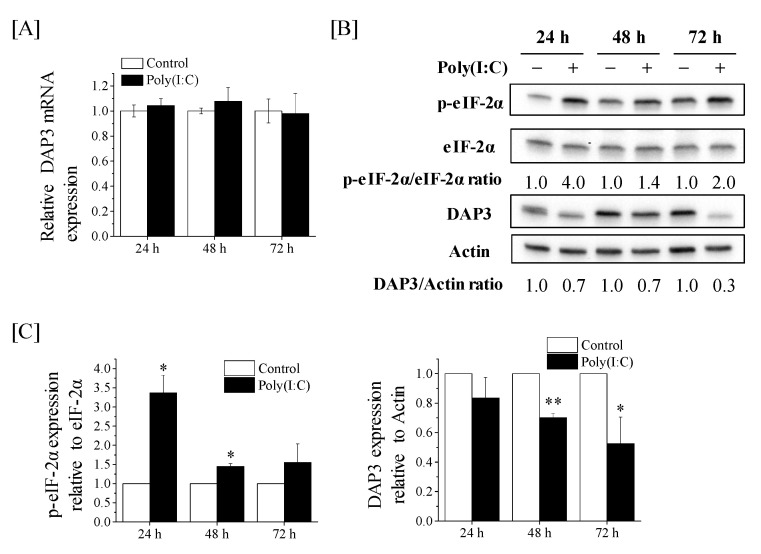
Effects of Poly(I:C) on the DAP3 mRNA expression and p-eIF-2α protein expression in A549 cells. (**A**) A549 cells were cultured with Poly(I:C) for 24–72 h and harvested for qRT-PCR. Data are presented as mean ± SD of three independent experiments. (**B**,**C**) A549 cells treated with Poly(I:C) were cultured for 24–72 h and harvested for western blotting. (**B**) Representative images of immunoblots are shown. Actin was used as the loading control. (**C**) The relative values of phosphorylated eukaryotic initiation factor-2α (p-eIF-2α)/eIF-2α and DAP3/actin ratio are presented as mean ± SD of three independent experiments. One sample *t*-test was performed using the GraphPad QuickCalcs. * *p* < 0.05, ** *p* < 0.01 versus control.

We finally explored the mechanism of Poly(I:C)-induced decrease in DAP3 protein expression of A549 cells. When the expression of DAP3 mRNA in A549 cells was analyzed using quantitative reverse transcription-polymerase chain reaction (qRT-PCR), no significant difference was noted in the DAP3 mRNA expression between the control cells and Poly(I:C)-treated cells (Figure 6A), suggesting that Poly(I:C) decreases DAP3 protein expression in a transcription-independent manner. It has been reported that double-stranded RNA induces the phosphorylation of eukaryotic initiation factor-2α (eIF-2α), which inhibits the translation of mRNA [21]. Therefore, we analyzed the effect of Poly(I:C) on the expression of phosphorylated eIF-2α (p-eIF-2α). As shown in Figure 6B,C, Poly(I:C) increased p-eIF-2α expression, followed by the downregulation of DAP3 protein expression.

## 3. Discussion

RLRs elicit immune responses against viruses and tumors through the mitochondria [22,23]. We previously reported that the RLR agonist Poly(I:C) enhanced radiosensitivity and that cotreatment with Poly(I:C) and IR more than additively increased cell death in human lung adenocarcinoma cells [15]. However, it remains unknown how Poly(I:C) modulates the cellular radiation response in human lung adenocarcinoma cells. Here we investigated the involvement of mitochondrial dynamics and the mitochondrial ribosome protein DAP3 in the modulation of the cellular radiation response by Poly(I:C). The present results demonstrate that Poly(I:C) decreased mitochondrial dynamics-related proteins and DAP3 protein expression. However, siRNA experiments revealed that DAP3, but not mitochondrial dynamics, regulates radioresistance and is involved in the more-than-additive effect of cotreatment with Poly(I:C) and IR on cell death in human lung adenocarcinoma cells.

Several studies have reported the relationship between mitochondrial dynamics and viral infection or RLR-mediated antiviral pathway [5,24,25,26]. For example, it has been reported that RLR activation induces the elongation of mitochondria [5]. In addition, dengue and human immunodeficiency viruses, which are detected by RLR [27,28], promote mitochondrial fusion by decreasing Drp1 expression [25,26]. These findings support our observations that Poly(I:C) reduces the Drp1 expression and induces mitochondrial elongation. Intriguingly, we observed a decrease in Mfn1 and L-OPA1 expressions following the downregulation of the Drp1 expression (Figure 1A,B). Saita et al. reported that the knockdown of Drp1 promotes the degradation of Mfn1 and L-OPA1 protein expression via the ubiquitin–proteosome systems and proteolytic cleavage, respectively [29]. Therefore, it is likely that the decrease in Mfn1 and L-OPA1 protein expressions resulted from the downregulation of Drp1 expression by Poly(I:C).

Although some reports indicate the involvement of Drp1 in cell death [16,30,31,32], this was not observed in A549 cells in this study. For example, Kobashigawa et al. reported that normal human fibroblast cells with Drp1-knockdown were resistant to gamma irradiation [30]. Similarly, Xu et al. showed that arenobufagin- or staurosporine-induced cell death was decreased by Drp1-knockdown in HCT116 human colon cancer cells [31]. These reports indicate that Drp1 mediates cell death. Conversely, the protective effects of Drp1 on cell death have also been reported [33,34,35]. For example, depletion of Drp1 is known to increase apoptosis in human colon cancer cells [33]. In addition, Chen et al. reported that silencing of Drp1 increased radiosensitivity of UM87MG and T98G human glioblastoma cells [34]. Furthermore, Akita et al. demonstrated that Drp1-knockdown sensitized A375 cells (a human malignant melanoma) and A549 to tumor necrosis factor-related apoptosis-inducing ligand, although Drp1-knockdown by itself did not increase the rate of apoptosis [35]. Therefore, taken together, it is likely that the involvement of Drp1 in cell death depends on the types of cell and stimulus-inducing cell death.

A pro-apoptotic effect of DAP3 has been reported [36,37,38]. DAP3 was originally identified as a mediator of IFN-gamma-induced cell death by functional gene cloning [36]. In addition, Miyazaki et al. showed that DAP3 expression was required for inducting anoikis, which is programmed cell death caused by loss of adhesion [37]. In contrast to these reports, Henning [19] and the current study suggest that DAP3 is related to radioresistance. Therefore, the role of DAP3 in cell death may depend on the type of stimulus inducing cell death.

Although this study depicted the involvement of DAP3 in radioresistance of human lung adenocarcinoma cells, the mechanism by which DAP3 regulates radioresistance remains unclear. DAP3 is known to control the mitochondria dynamics as well as the mitochondrial function. Xiao et al. reported that the knockdown of DAP3 increased the fragmentation in mitochondria [39]. We also observed fragmented mitochondria in DAP3-knockdown A549 cells (Figure S3). Since several reports have demonstrated an association between mitochondria fission and cell death [16,30,31,32,40,41], we assumed that mitochondrial fragmentation contributes to the increase in IR-induced cell death via DAP3-knockdown. However, Mfn1-knockdown leading to mitochondrial fragmentation did not affect the IR-induced cell death in A549 cells (Figure 1D and Figure S2). Therefore, it is likely that DAP3 controls the radioresistance of lung adenocarcinoma cells in a mitochondria fission-independent manner. Mitochondrial functions, such as energy metabolism, are closely related to the radioresistance of most cancers, including lung cancer [42,43]. Since DAP3 is essential for maintaining mitochondrial functions, including ATP production [39], it is possible that DAP3-knockdown causes radiosensitization through impairment of mitochondrial functions, such as energy metabolism. Further studies focusing on mitochondrial function are needed to clarify DAP3-mediated radioresistance mechanisms.

As mentioned earlier, the fragmentation of mitochondria was observed in DAP3-knockdown A549 cells (Figure S3). Interestingly, Poly(I:C) treatment induced mitochondrial elongation despite the downregulation of DAP3 protein expression (Figure 1C). Xiao et al. reported that DAP3-knockdown increased the fragmentation of mitochondrial through phosphorylation of Drp1 [39]. Considering that Poly(I:C) reduces not only DAP3 but also Drp1 protein expression and that the mitochondria morphology of Poly(I:C)-treated cells is similar to that of Drp1-knockdown cells, it seems that the mitochondrial morphology of Poly(I:C)-treated A549 cells is predominantly regulated by the downregulation of the Drp1 expression.

Here, we showed that DAP3-knockdown increased cell death after IR (Figure 4). Notably, DAP3-knockdown did not affect the cell death induced by cotreatment with Poly(I:C) and IR (Figure 5). These results suggest that the effect of Poly(I:C) to increase IR-induced cell death is related to DAP3, because if the effect is independent of DAP3, DAP3-knockdown should further increase cell death induced by cotreatment with Poly(I:C) and IR. Since Poly(I:C) decreased DAP3 protein expression, it is believed that Poly(I:C) increases IR-induced cell death through the downregulation of DAP3 expression.

The present results suggest that downregulation of DAP3 protein expression by Poly(I:C) participated in the more-than-additive effect of cotreatment on cell death. To the best of our knowledge, this is the first study to suggest that an RLR agonist negatively regulates DAP3 protein expression. Interestingly, Poly(I:C) post-transcriptionally decreased DAP3 protein expression (Figure 6). Since Poly(I:C) increased the p-eIF-2α expression followed by the downregulation of DAP3 protein expression, it is believed that Poly(I:C) decreased DAP3 protein expression through inhibition of translation of DAP3 mRNA. Indeed, we could not exclude the possibility that mtDNA was involved in the post-transcriptional regulation of DAP3 expression by Poly(I:C). A previous report suggests that the amount of mtDNA controls DAP3 protein expression [44] and that infection with human immunodeficiency virus 1, which is detected by RLR [27], decreases mtDNA [45]. Further study of mtDNA will be required to investigate this possibility.

In conclusion, we show that the downregulation of DAP3 protein expression by Poly(I:C) contributes to the more-than-additive effect of cotreatment with Poly(I:C) and IR on cell death in human lung adenocarcinoma cells. In addition, the present results highlighting the importance of DAP3 in the cellular radiation response of human lung adenocarcinoma cells improve our understanding of DAP3-mediated radioresistance mechanisms and have implications on the efficacy of radiation therapy for lung adenocarcinoma.

## 4. Materials and Methods

### 4.1. Reagents

Calcium- and magnesium-free phosphate-buffered saline was purchased from Wako Pure Chemical Industries, Ltd. (Osaka, Japan). PI was purchased from Sigma-Aldrich (Merck KGaA, Darmstadt, Germany). Poly(I:C)-HMW/LyoVec™ (Poly(I:C)), which is a complex of the synthetic double-stranded RNA analog poly(I:C) and a transfection reagent (LyoVec™), were purchased from InvivoGen (San Diego, CA, USA). Annexin V-FITC was purchased from BioLegend, Inc. (San Diego, CA, USA). Anti-rabbit horseradish peroxidase (HRP)-conjugated IgG and anti-mouse HRP-conjugated IgG secondary antibodies, anti-Mfn1 (cat. no. 14739), anti-OPA1 (cat. no. 80471), anti-Drp1 (cat. no. 5391), anti-eIF-2α (cat. no. 9722), anti-phospho-eIF-2α (cat. no. 3597), anti-β-actin (cat. no. 4967) monoclonal antibodies, and SignalSilence^®^ Mfn1 (cat. no. 13303) siRNA were purchased from Cell Signaling Technology Inc. (Danvers, MA, USA). Anti-DAP3 (cat. no. 610662) monoclonal primary antibody was purchased from BD Biosciences (Franklin Lakes, NJ, USA). Ambion Silencer^®^ Select Pre-designed siRNA against the gene-encoding Drp1 (cat. no. s19560), the gene-encoding DAP3 (cat. no. s1506), and Silencer^®^ Select Negative #1 Control (cat. no. AM4611) siRNAs were purchased from Thermo Fisher Scientific, Inc. (Waltham, MA, USA).

### 4.2. Cell Culture and Treatment

Human lung adenocarcinoma cells A549 and H1299 were purchased from Riken Bio-Resource Center (Tsukuba, Japan) and American Type Culture Collection (ATCC, Manassas, VA, USA), respectively. A549 cells were maintained in Dulbecco’s modified Eagle’s medium (Sigma-Aldrich) supplemented with 1% penicillin/streptomycin (Wako Pure Chemical Industries, Ltd.) and 10% heat-inactivated fetal bovine serum (Sigma-Aldrich) at 37 °C in a humidified atmosphere of 5% CO_2_. H1299 cells were maintained in RPMI1640 medium (Gibco^®^; Invitrogen/Thermo Fisher Scientific, Waltham, MA, USA) supplemented with 1% penicillin/streptomycin and 10% heat-inactivated FBS at 37 °C in a humidified atmosphere of 5% CO_2_.

Cells were seeded onto 35-mm culture dishes (6.0 × 10^4^ cells) or 60-mm culture dishes (1.2 × 10^5^ cells) (Sumitomo Bakelite Co., Ltd., Tokyo, Japan) and were cultured for 6 h to allow adherence. After incubation, the RLR agonist Poly(I:C) (250 ng/mL) was added to the culture medium for the indicated time periods. Next, the cells were harvested using 0.1% trypsin-ethylenediaminetetraacetic acid (Wako Pure Chemical Industries, Ltd.) for subsequent analysis. In some experiments, X-ray irradiation was performed 1 h after Poly(I:C) administration, and the treated cells were cultured.

### 4.3. In Vitro X-ray Irradiation

Cells were irradiated (150 kVp; 20 mA; 0.5-mm Al filter and 0.3-mm Cu filter) using an X-ray generator (MBR-1520R-3; Hitachi, Ltd., Tokyo, Japan) at a distance of 45 cm from the focus and a dose rate of 0.99–1.02 Gy/min.

### 4.4. SDS-PAGE and Western Blotting

SDS-PAGE and western blot analysis were performed as previously reported [46]. The following primary antibodies were used: anti-Mfn1 (1:3000), anti-OPA1 (1:3000), anti-Drp1 (1:3000), anti-DAP3 (1:3000), and anti-β-actin (1:4000). The following secondary antibodies were used: HRP-conjugated anti-rabbit IgG (1:10,000) and HRP-conjugated anti-rabbit IgG (1:10,000). The antigens were visualized using the Clarity^TM^ Western ECL Substrate (Bio-Rad Laboratories, Inc., Hercules, CA, USA). Blot stripping was performed using Stripping Solution (Wako Pure Chemical Industries, Ltd.). Quantification of the bands was performed using ImageJ software (National Institutes of Health, Bethesda, MD, USA).

### 4.5. Mitochondrial Morphology

Cells were seeded onto 35-mm glass bottomed dishes (6.0 × 10^4^ cells) and cultured for 3 days. In an experiment, the cells were cultured in the presence of 250 ng/ml Poly(I:C). After culturing for 3 days, the cells were stained with 100 nM MitoTracker^TM^ Green FM (Invitrogen; Thermo Fisher Scientific, Inc.) for 30 min at 37 °C in a humidified atmosphere of 5% CO_2_. After washing with medium, fresh growth medium was supplied. Fluorescence images were obtained using Olympus IX71 fluorescent microscope (Tokyo, Japan) and DP2-BSWsoftware (Olympus).

### 4.6. Quantitative Reverse Transcription Polymerase Chain Reaction (qRT-PCR)

Total RNA extraction and synthesis of complementary DNA templates were performed as previously described [47]. The synthesis of complementary DNA templates was performed using an iScript cDNA synthesis kit (Bio-Rad Laboratories, Inc.) according to the manufacturer’s instructions. Quantitative RT-PCR was performed using Power SYBR^®^ Green Master Mix (Applied Biosystems Inc., Carlsbad, CA, USA) in a Step One Plus™ system (Applied Biosystems Inc.). Differences in gene expression relative to unirradiated controls were determined using ΔCt values after normalization to the housekeeping gene β-actin. β-actin primer sequences are reported elsewhere [48]. Primer sequences for DAP3 were 5′-AGGAGTTGCTGGGAAAGGA-3′ (sense) and 5′-TGGAAACCAGGATGGGAATA-3′ (antisense).

### 4.7. siRNA Transfection

Cells were transfected with siRNA targeting Drp1, Mfn1 or Control siRNA using Lipofectamine^®^ RNAiMAX (Invitrogen; Thermo Fisher Scientific, Inc.) according to the manufacturer’s protocol. Following incubation for 48 h, Drp1 and Mfn1 siRNA transfected cells were harvested and used for subsequent analyses. Transfections of siRNA targeting either DAP3 or Control siRNA were performed twice. In brief, cells transfected for 48 h were harvested, transfected again, and cultured for another 48 h. After the second transfection, the cells were harvested and used for subsequent analyses. The final concentration of all siRNAs was 10 nM.

### 4.8. Detection of Cell Death

Cell death was analyzed by annexin V-FITC and PI staining as previously reported [49]. In brief, treated cells were harvested, washed, and suspended in annexin V Binding Buffer (BioLegend). Annexin V-FITC (2.5 µg/mL) and PI (50 µg/mL) solutions were added to cell suspensions and incubated for 15 min at room temperature in the dark. The cells were then analyzed using flow cytometry (Cytomics FC500; Beckman–Coulter, Fullerton, CA, USA).

### 4.9. Clonogenic Survival Assay

DAP3-knockdown cells were seeded onto 35-mm culture dishes (6.0 × 10^4^ cells) and incubated for 6 h to allow them to adhere to the dish. After incubation, the cells were exposed to X-rays and cultured for about 20 h. The cultured cells were harvested using 0.1% trypsin-ethylenediaminetetraacetic acid and seeded onto 60-mm culture dishes. The cells were incubated for 7–12 days, fixed with methanol, and stained with Giemsa solution (Wako Pure Chemical Industries, Ltd.). Experiments were performed in triplicate. Colonies containing > 50 cells were counted. The surviving fraction at each radiation dose was calculated as previously reported [15].

### 4.10. Statistical Analysis

Data are presented as the mean ± standard deviation of three independent experiments. Comparisons between the control and experimental groups were performed using the two-sided Student’s *t*-test or Mann–Whitney U-test depending on data distribution. *p* values < 0.05 were used to indicate statistically significant differences. Excel 2016 software (Microsoft, Washington, DC, USA) along with the add-in software Statcel 4 (The Publisher OMS Ltd., Tokyo, Japan) was used to perform statistical analyses. When control group is considered as 100%, one sample *t* test was performed using GraphPad QuickCalcs (see “URLs”).

### 4.11. URLs

GraphPad QuickCalcs, https://www.graphpad.com/quickcalcs/.

## Supplementary Materials

The following are available online at https://www.mdpi.com/1422-0067/22/1/420/s1.

## Abbreviations

DAP3	Death-associated protein 3
Drp1	Dynamin-related protein 1
eIF-2α	eukaryotic initiation factor-2α
HRP	Horseradish peroxidase
IFNs	Interferons
IR	Ionizing radiation
L-OPA1	long isoform OPA1
mtDNA	Mitochondrial DNA
Mfn1/2	Mitofusin-1/2
OPA1	Optic atrophy 1
p-eIF-2α	Phosphorylated eIF-2α
Poly(I:C)	Poly(I:C)-HMW/LyoVec™
PI	Propidium iodide
qRT-PCR	Quantitative reverse transcription polymerase chain reaction
RIG-I	Retinoic acid-inducible gene-I
RLRs	RIG-I-like receptors
