# Supplementary material for: DAP3 Is Involved in Modulation of Cellular Radiation Response by RIG-I-Like Receptor Agonist in Human Lung Adenocarcinoma Cells
Source: Int J Mol Sci. 2021 Jan 3;22(1):420. doi: 10.3390/ijms22010420 (PMC7795940; doi:10.3390/ijms22010420)
Supplement: Supplementary file 1 [file ijms-22-00420-s001.pdf]

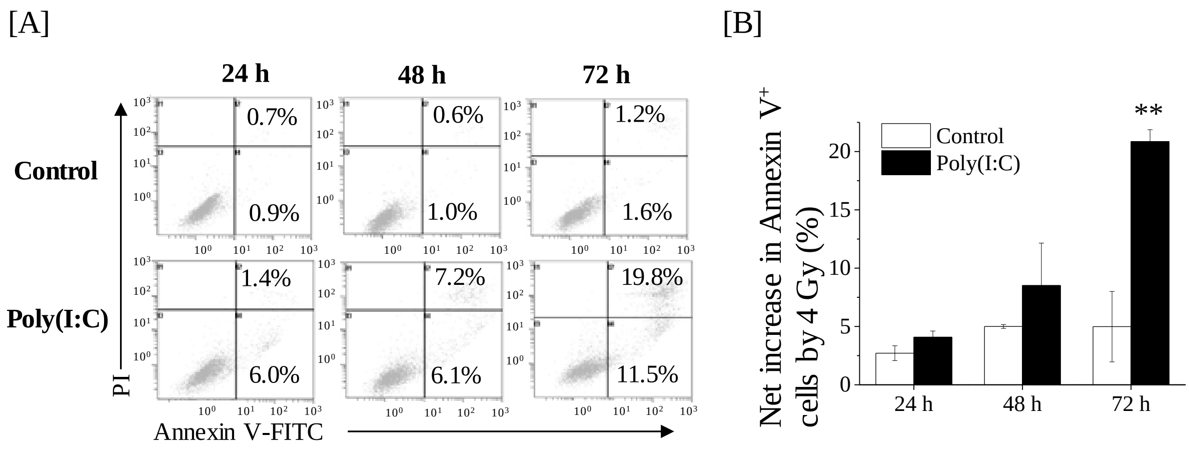

Figure S1. Effects of Poly(I:C) to induce cell death in A549 cells. (**A**) A549 cells were cultured for 24, 48 and 72 h in the presence of 250 ng/ml Poly(I:C). After culturing, the cells were harvested for cell death assay using annexin V/PI staining. Representative cytograms of annexin V/PI staining are shown. The inset numbers indicate the fractions of annexin V+/PI− or annexin V+/PI+ cells. **(B)** A549 cells were incubated with Poly(I:C). After incubation for 1 h, the cells were irradiated with 4 Gy. After culturing for 24, 48 and 72 h, the cells were harvested for cell death assay using annexin V/PI staining. The results are presented as the net increase in the fraction of annexin V+ cells (the sum of annexin V+/PI− cells and annexin V+/PI+ cells) by 4 Gy. Data are mean ± SD of three independent experiments. **p < 0.01 versus control.

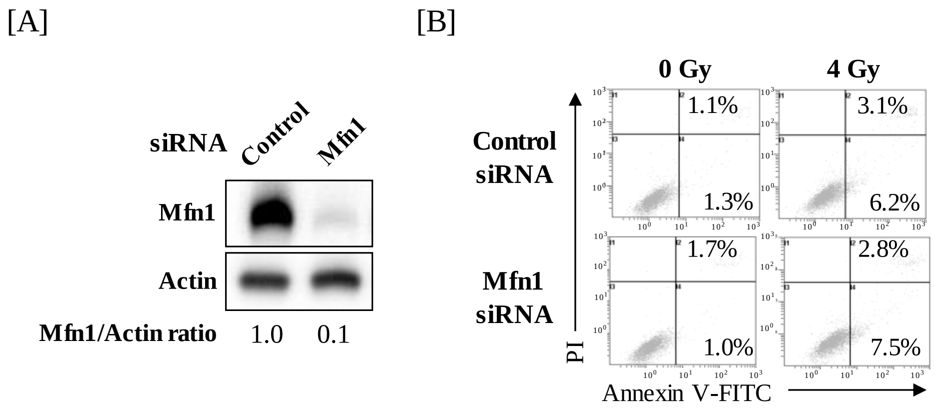

Figure S2. Effects of Mfn1-knockdown on IR-induced cell death in A549 cells. (**A**) A549 cells transfected with control or Mfn1 siRNA were harvested, and the Mfn1 protein expression was analyzed by western blotting. Representative images of immunoblots are shown. Actin was used as a loading control. The relative values of Mfn1/actin ratio are presented. (**B**) Mfn1-knockdwon A549 cells were treated with 4 Gy. After culturing for 72 h, the cells were harvested for cell death analysis using annexin V-FITC/PI staining. Representative cytograms of annexin V/PI staining are shown. The inset numbers indicate the fractions of annexin V+/PI− or annexin V+/PI+ cells.

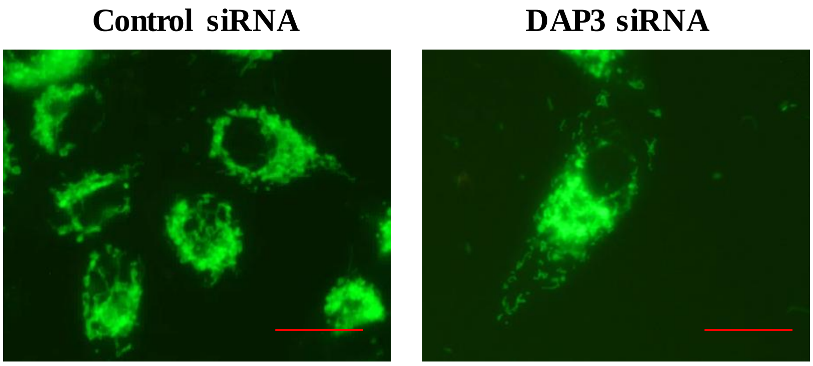

Figure S3. Mitochondrial morphology of DAP3-knocodown A549 cells. A549 cells transfected with control or DAP3 siRNA were cultured for 48 h and harvested for mitochondrial morphology analysis. Scale bar = 20 μm.
